# Advanced cholangiocarcinoma with human epidermal growth factor receptor 2 (HER2) amplification treated with Trastuzumab deruxtecan (T-DXd): A case report

**DOI:** 10.1097/MD.0000000000044094

**Published:** 2025-08-29

**Authors:** Xiaohui Bao, Zhi Chen, Jin Xiong, Zhenzhou Yang, Ni Zhang

**Affiliations:** a Department of Cancer Center, The Second Affiliated Hospital of Chongqing Medical University, Chongqing, China.

**Keywords:** backline treatment, case report, Cholangiocarcinoma (CCA), HER2 amplification, Trastuzumab deruxtecan

## Abstract

**Rationale:**

Human epidermal growth factor receptor 2 (HER2)-positive cholangiocarcinoma is a rare disease with a low incidence and high degree of malignancy. Trastuzumab deruxtecan (T-DXd) has been approved for the treatment of HER2-positive breast and gastric cancer. However, it is still in the initial exploration period for HER2-positive cholangiocarcinoma.

**Patient concerns:**

A 57-year-old Han Chinese male patient with recurrent metastatic cholangiocarcinoma who was tested for HER2 expression in surgical specimens in the absence of reliable drug therapy.

**Diagnoses:**

Postoperative pathological examination confirmed a diagnosis of moderately to poorly differentiated adenocarcinoma of the common bile duct.

**Interventions:**

Our case revealed HER2 amplification and effectively received T-DXd as backline therapy.

**Outcomes:**

After approximately 4 months without disease progression, the patient experienced an increase in plasma tumor markers; however, he regained disease control after receiving T-DXd in combination with lenvatinib with a favorable physical status and quality of life.

**Lessons:**

Reexamination of tumor tissue samples to identify target mutations is necessary for backline treatment of cholangiocarcinoma. T-DXd is effective in the treatment of HER2 amplification cholangiocarcinoma and is relatively well tolerated after multiple lines of therapy. The combination of multi-target tyrosinase inhibitors is a possible strategy for overcoming resistance in the future.

## 1. Introduction

Biliary tract carcinoma (BTC) is a rare and aggressive malignancy that originates from the epithelium of the bile ducts and encompasses intrahepatic cholangiocarcinoma (iCCA), extrahepatic cholangiocarcinoma (eCCA), gallbladder cancer (GBC), and cancer of the ampulla of Vater. Collectively, these cancers account for approximately 15% of all primary liver cancers and 3% of gastrointestinal malignancies.^[1]^ Alarmingly, the incidence and mortality rates of BTC are increasing globally. Most patients with cholangiocarcinoma are diagnosed at an advanced stage, with either locally advanced or metastatic disease, leading to poor prognosis. Only 10% to 40% of patients are eligible for surgical resection.^[2]^ Currently, surgery followed by adjuvant capecitabine therapy is the only curative treatment option. Unfortunately, most patients who undergo radical surgery experience local recurrence or distant metastases. Historically, the standard treatment for advanced BTC has been the combination of gemcitabine and cisplatin.^[3]^ Recently, the TOPAZ study demonstrated the benefits of combining immunotherapy with chemotherapy.^[4]^ However, immunological predictors of response are infrequent, and there is a notable lack of reliable biomarkers.^[5]^ Therefore, diverse perspectives and improved treatment options are urgently needed.

Recently, the advent of targeted therapies has opened new avenues for the treatment of various malignancies. Notably, the therapeutic efficacy of anti-HER2 drugs in breast^[6]^ and gastric^[7]^ cancers, both characterized by high rates of human epidermal growth factor receptor 2 (HER2) overexpression, has led to a growing interest in the application of anti-HER2 therapy for HER2-overexpressing cholangiocarcinoma, particularly in eCCA and GBC. The HER2 is a tyrosine kinase transmembrane receptor that serves as a promising biomarker for targeted therapy, with its overexpression and gene amplification implicated in tumor growth and progression.^[8]^ Reports indicate that HER2 overexpression, gene amplification, or both are present in 15% to 30% of GBC cases, 10% to 20% of eCCA cases, and 3% to 5% of iCCA cases.^[9]^ A systematic review and meta-analysis reported a significantly higher rate of HER2 expression in eCCA (19.9%) than in iCCA (4.8%) (*P* = .0049).^[10]^ Additionally, it has been reported that approximately 3.6% of patients with eCCA exhibit HER2 amplification.^[11]^ However, there is a paucity of prospective trial data on the therapeutic targeting of HER2 in BTCs. To date, there have been few documented cases of cholangiocarcinoma treated with anti-HER2 therapy. Therefore, this report presents the first real-world evidence of the efficacy and safety of Trastuzumab deruxtecan (T-DXd), an antibody-drug conjugate (ADC) composed of a humanized monoclonal anti-HER2 antibody, for the treatment of advanced cholangiocarcinoma and management of subsequent drug resistance.

## 2. Case description

**Figure 1.**
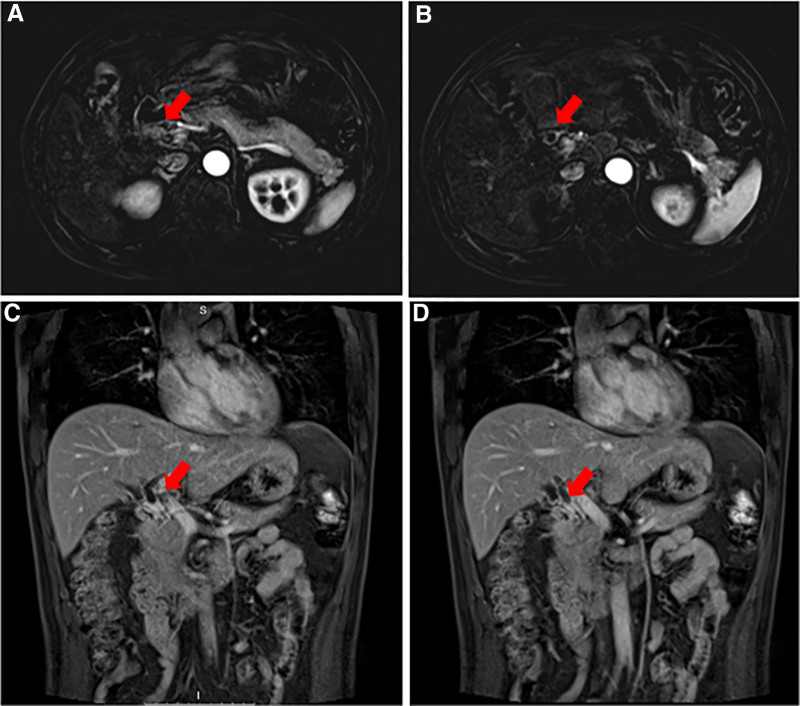
Axial (A and B) and coronal (C and D) views at different levels. Magnetic resonance cholangiopancreatography showed segmental thickening of the upper part of the common bile duct.

A 57-year-old Han Chinese male was admitted to the Second Affiliated Hospital of Chongqing Medical University on November 21, 2019, complaining of abdominal pain. He had no notable personal medical or psychosocial history, and no family history of malignancy. Magnetic resonance imaging revealed segmental thickening of the upper portion of the common bile duct, with the tumor being a possible diagnosis (Fig. 1). Subsequently, the patient underwent “radical cholangiocarcinoma surgery, choledochotomy, and biliary anastomosis” for cholangiocarcinoma and Roux-en-Y hepatic-jejunal end-side anastomosis was used without hepatectomy. Postoperative recovery was uneventful, with significant relief of the abdominal pain.

**Figure 2.**
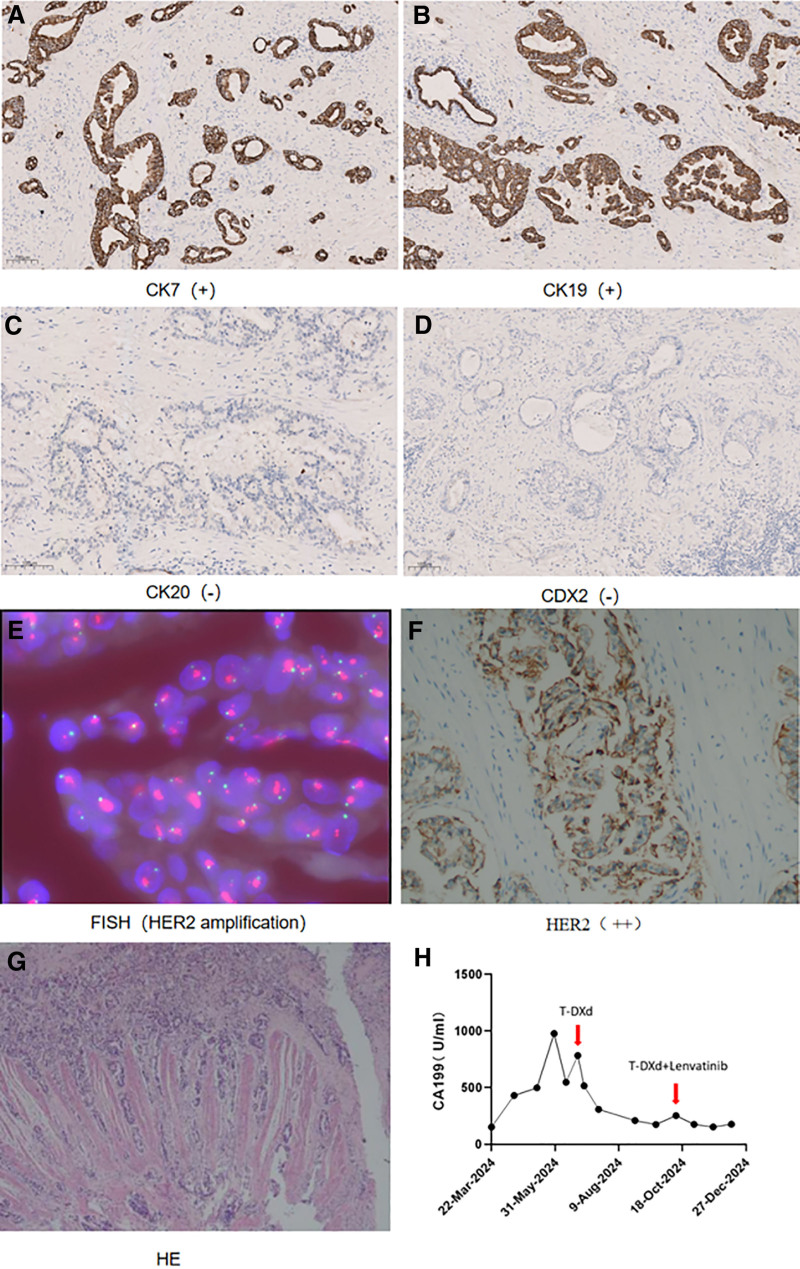
(A–D) IHC respectively showed CK7(+), CK19(+), CK20(−), CDX2(−); (E, F) IHC showed HER2(++) and FISH showed HER2 amplification; (G) H&E showed adenocarcinoma of the common bile duct; (H) A line chart showing the trend of CA199 in response to posterior line therapy; H&E = hematoxylin and eosin staining; HER2 = human epidermal growth factor receptor 2; IHC = immunohistochemistry; FISH = fluorescence in situ hybridization.

Postoperative pathological examination confirmed a diagnosis of moderately to poorly differentiated adenocarcinoma of the common bile duct, which had infiltrated the entire thickness of the duct wall. Notably, there was evidence of nerve invasion but no vascular tumor thrombus or malignancy at the resection margins, and lymph node involvement was absent (0/3). Immunohistochemistry (IHC) suggested CK7(+), CK19(+), CK20(−), CDX2(−), thus excluding a gastrointestinal origin (Fig. 2). Genetic testing of the tissue identified a TP53 mutation along with expression of VEGFR1 and VEGFR2 proteins. The patient’s condition stabilized during regular postoperative follow-up.

Two years later, in December 2021, the patient revisited our hospital due to jaundice. Enhanced magnetic resonance imaging of the upper abdomen indicated mild dilatation of the intrahepatic bile ducts and a suspicious mass at the confluence of the right and left hepatic ducts, suggestive of cholangiocarcinoma recurrence. Disease-free survival was recorded at 2 years. Consequently, the patient underwent a second radical surgery for cholangiocarcinoma on January 4, 2022. The patient subsequently received a regimen of gemcitabine combined with cisplatin and bevacizumab until August 2022, and maintained a stable condition during follow-up.

However, a chest and abdominal computed tomography (CT) scan on June 5, 2023, revealed enlargement of the anterolateral lymph nodes in the gastric sinusoids, marking disease progression, with a progression-free survival 1 (PFS1) of 17 months. Consequently, the patient was treated with a regimen of durvalumab 200 mg, albumin paclitaxel 400 mg, and carboplatin 600 mg, administered every 3 weeks for 4 cycles from June 15, 2023, to August 20, 2023. Additionally, the patient received radiation therapy targeting the abdominal lesion, with a planned treatment volume of 45 Gy over 25 fractions, followed by maintenance therapy with durvalumab (200 mg) for 8 cycles.

Unfortunately, a CT scan in April 2024 revealed new omental and peritoneal lymph node metastases, and PFS2 was registered for 10 months. As a result, the patient was administered a combination of cadonilimab 500 mg, oxaliplatin 230 mg, and capecitabine for 2 cycles from April 18, 2024 to May 31, 2024. However, severe hand and foot reactions necessitated the cessation of chemotherapy. A follow-up CT scan on May 31, 2024, showed multiple nodules within the omentum, peritoneum, and mesentery, with some nodules slightly enlarged, and the carbohydrate antigen (CA) 199 level increased from 497 to 977 U/mL (normal range: 0–37 U/mL). PFS3 was recorded at 2 months.

**Figure 3.**
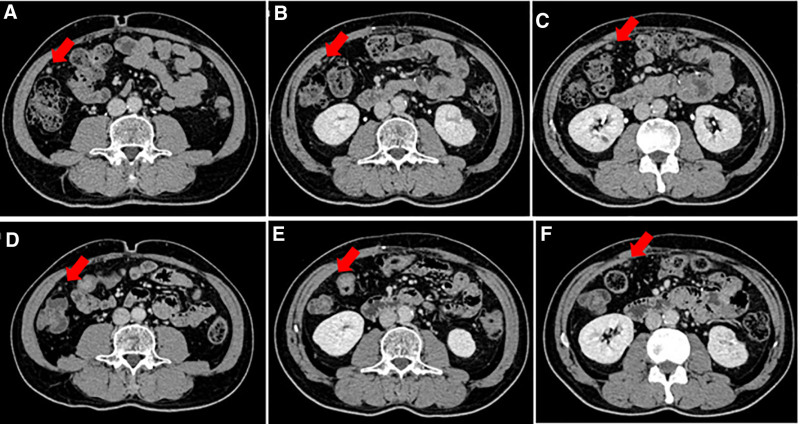
CT showed partial response of the disease, before the use of T-DXd (A–C) and after 2 cycles of T-DXd treatment (D–F); CT = computed tomography; T-DXd = Trastuzumab deruxtecan.

Considering the individualization principle of backline therapy, HER2 expression was evaluated in the second surgical specimens, confirming positive expression via IHC and fluorescence in situ hybridization assays (Fig. 2). Consequently, the patient commenced treatment with T-DXd at a dose of 5.4 mg/kg administered every 3 weeks beginning in June 2024. A follow-up abdominal enhanced CT on July 15, 2024, indicated partial shrinkage of the metastatic lesions, with CA 199 levels declining from 783 to 174 U/mL (Figs. 2 and 3).

**Figure 4.**
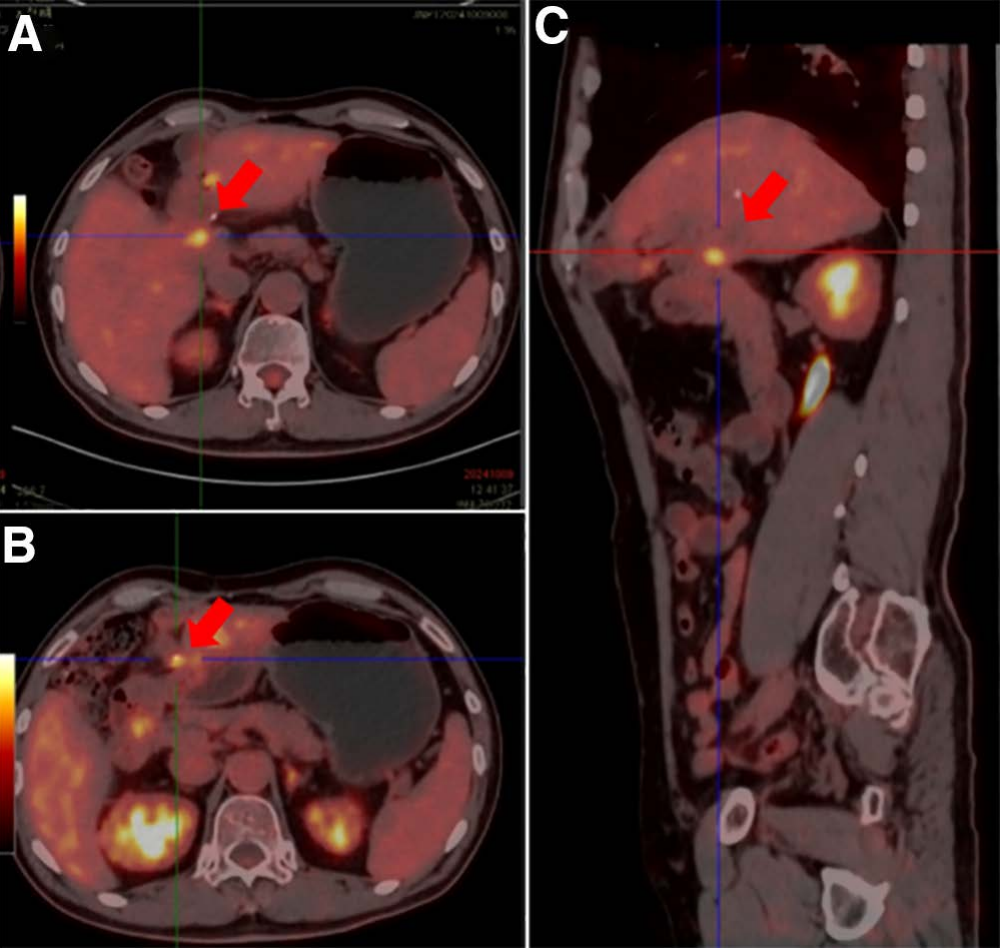
Axial (A and B) and sagittal (C) views at different levels. The positron emission tomography/computed tomography showed that the metastases were still showing tumor activity.

During T-DXd treatment, the patient experienced grade 2 myelosuppression characterized by leukopenia and anemia, along with grade 1 abnormal liver function. All adverse effects were managed using symptomatic treatment. However, CA 199 levels subsequently increased from 174 to 253 U/mL after 4 months of T-DXd therapy. Positron emission tomography-computed tomography in October 2024 revealed ongoing tumor activity in the metastases (Fig. 4).

Following the onset of T-DXd resistance, the treatment plan was modified to include a multi-target tyrosine kinase inhibitor (TKI), lenvatinib (8 mg qd), in combination with T-DXd. This strategy resulted in renewed disease control, with only grade 1 thrombocytopenia being observed. The patient continues to be under follow-up care, maintaining low CA 199 levels and good quality of life.

## 3. Discussion

In this report, we present a compelling case of postoperative recurrence of cholangiocarcinoma that exhibited HER2 amplification, a condition for which suitable backline treatment options are lacking. Remarkably, T-DXd administration resulted in improved disease control. To our knowledge, cases of cholangiocarcinoma with HER2 amplification are rare in sporadic cases. This report highlights the significance of HER2 amplification in the treatment of cholangiocarcinoma, and emphasizes the efficacy of T-DXd along with alternative therapeutic options following resistance.

The prognosis of patients with advanced cholangiocarcinoma is often poor due to their relatively short life expectancy, underscoring the critical need for innovative treatment strategies. Reports indicate that approximately 40% of cholangiocarcinoma cases harbor genetic alterations that can be targeted by specific therapies.^[1]^ The prevalent molecular alterations include isocitrate dehydrogenase 1 mutations (15%–20%), fibroblast growth factor receptor 2 (FGFR2) fusions (10%), HER2 amplifications and mutations (5%–10%), and proto-oncogene BRAF mutations (3%).^[2]^ Consequently, various drugs targeting different mutational profiles are being explored for the treatment of advanced cholangiocarcinoma. For instance, pemigatinib has received approval for the management of advanced cholangiocarcinoma because of its potent activity as a selective oral small-molecule inhibitor of FGFR1, FGFR2, and FGFR3.^[12]^

In contrast, HER2 represents a promising target for molecularly targeted therapy of cholangiocarcinoma. HER2 is a member of a family of 4 proteins: HER1 (also known as the epidermal growth factor receptor), HER3, and HER4. HER2 overexpression can occur across various solid tumor types, including breast, gastric, biliary tract, bladder, pancreatic, and gynecological cancers. HER2 overexpression has been linked to a more aggressive tumor phenotype, poor prognosis, increased risk of recurrence, and limited response to conventional chemotherapy. In our case of advanced cholangiocarcinoma, it was crucial to assess HER2 protein expression in the second surgical specimen following failure of standard treatment. Although HER2 genetic alterations were absent in the patient’s initial surgical specimen, we suspected that this could largely result from the inherent heterogeneity of cholangiocarcinoma.

ADCs are sophisticated therapeutics that fuse monoclonal antibodies to a cytotoxic chemotherapy payload, allowing for the targeted delivery of chemotherapy to cancer cells while potentially mitigating systemic toxicity. ADCs constitute a promising treatment avenue across various cancer types, and remain an important focus of ongoing research and development.^[13]^ T-DXd is an ADC composed of a humanized monoclonal anti-HER2 antibody, cleavable tetrapeptide-based linker, and potent topoisomerase I inhibitor. Currently, T-DXd is approved for use in advanced breast cancer with low or overexpression of HER2, HER2-positive gastric cancer, and non-small cell lung cancer.^[14]^ Few clinical studies have assessed the application of T-DXd in cholangiocarcinoma. The Phase II HERB trial evaluated T-DXd in patients with unresectable or recurrent BTC. Patients exhibiting IHC 3+ and 2+ (HER2-positive patients) demonstrated an objective response rate (ORR) of 36.4%, a disease control rate of 81.8%, a median PFS of 5.1 months (95% CI, 3.0–7.3), and a median overall survival (OS) of 7.1 months (95% CI, 4.7–14.8).^[15]^ Moreover, the response rate exceeded that of a clinical trial investigating another HER2-targeted combination therapy involving trastuzumab and pertuzumab.^[16]^ Further exploration of T-DXd was conducted in the phase II DESTINY-PanTumor02 trial across 7 tumor cohorts, where the BTC cohort exhibited an ORR of 22.0%, with a median PFS and OS of 4.6 months (95% CI, 3.1–6.0) and 7.0 months (95% CI, 4.6–10.2), respectively.^[17]^ Notably, patients classified as IHC 3+ exhibited superior outcomes compared with those categorized as IHC 2+ (ORR, 56.3%; median OS, 12.4 months). In our patient, T-DXd was administered as a fourth-line treatment, achieving a PFS of 4 months, which is consistent with the findings of the DESTINY-PanTumor02 trial.^[17]^ However, it is important to acknowledge that T-DXd is associated with unique potential side effects, some of which may be serious or fatal. Vigilant monitoring is essential, as reported incidence rates for interstitial lung disease and cardiotoxicity were 10.9% and 1.2%, respectively, in a meta-analysis of 8 clinical trials.^[18]^ Additionally, common grade ≥ 3 treatment-related adverse events included anemia (53.1%), neutropenia (31.3%), decreased white blood cell count (31.3%), and decreased lymphocyte count (21.9%), with 25.0% of patients developing interstitial lung disease. Our patient experienced similar adverse effects, including leukopenia, anemia, neutropenia, and liver impairment, although these were limited to grade 1 to 2.

Despite the significant advancements in ADC technology, resistance remains a formidable challenge. Numerous mechanisms driving ADC resistance have been identified, including the following 8 aspects: antigen-related resistance, payload-related resistance, impaired internalization and trafficking pathways, dysfunctional lysosomal function, drug-efflux pump activity, alterations in the cell cycle, activation of alternative signaling pathways, and dysregulation of apoptotic mechanisms.^[19]^ Notably, one common mechanism of resistance involves increased drug extrusion via overexpression of drug efflux pumps. Development of strategies to overcome ADC resistance is an ongoing and complex area of research, whether through new ADC designs or combination therapies. Another critical concern is tumor heterogeneity, which may reduce the efficacy of ADCs against tumor cells exhibiting low antigen expression. To counteract resistance, combination therapies, such as TKI plus T-DXd, have been evaluated in multiple phase I, II, and III trials to explore potential synergistic effects. The augmentation of ADCs with immune checkpoint inhibitors may enhance CD8+ effector T-cell infiltration in tumors, thereby improving therapeutic responses.^[20]^ In our case, the combination of the multi-target TKI lenvatinib with T-DXd proved effective against T-DXd resistance, ultimately prolonging patient survival. We hypothesized that the development of T-DXd resistance in this patient may be linked to the activation of downstream signaling pathways.

## 4. Conclusions

In summary, T-DXd demonstrated efficacy and safety as a backline treatment for HER2-positive cholangiocarcinoma. Retesting genetic or tissue samples should be emphasized to guide treatment decisions, and the combination of T-DXd and TKIs can provide a viable option following resistance to T-DXd therapy.

## Acknowledgments

The authors thank the patient and his family for granting permission to publish this case report.

## Author contributions

**Supervision:** Zhi Chen.

**Validation:** Zhi Chen.

**Writing – original draft:** Xiaohui Bao, Jin Xiong.

**Writing – review & editing:** Zhenzhou Yang, Ni Zhang.
